# Metabolic milieu associates with impaired skeletal characteristics in obesity

**DOI:** 10.1371/journal.pone.0179660

**Published:** 2017-06-22

**Authors:** Heli T. Viljakainen, Heikki A. Koistinen, Taina Tervahartiala, Timo Sorsa, Sture Andersson, Outi Mäkitie

**Affiliations:** 1 Children's Hospital, University of Helsinki and Helsinki University Hospital, Helsinki, Finland; 2 Folkhälsan Research Centre, Helsinki, Finland; 3 Department of Medicine and Abdominal Center: Endocrinology, University of Helsinki and Helsinki University Hospital, Helsinki, Finland; 4 Minerva Foundation Institute for Medical Research, Helsinki, Finland; 5 Department of Oral and Maxillofacial Diseases, University of Helsinki and Helsinki University Hospital, Helsinki, Finland; 6 Division of Periodontology, Department of Dental Medicine, Karolinska Institutet, Huddinge, Sweden; 7 Center for Molecular Medicine, Karolinska Institutet, and Clinical Genetics, Karolinska University Hospital, Stockholm, Sweden; Charles P. Darby Children's Research Institute, 173 Ashley Avenue, Charleston, SC 29425, USA, UNITED STATES

## Abstract

High leptin concentration, low-grade inflammation, and insulin resistance often coexist in obese subjects; this adverse metabolic milieu may be the main culprit for increased fracture risk and impaired bone quality seen in patients with type 2 diabetes. We examined the associations of leptin, hs (high sensitivity)- CRP and insulin resistance with bone turnover markers (BTMs) and bone characteristics in 55 young obese adults (median BMI 40 kg/m^2^) and 65 non-obese controls. Mean age of the subjects was 19.5 ± 2.5 years (mean ± SD). Concentrations of leptin, adiponectin, hs-CRP, MMP-8 and TIMP-1, fasting plasma glucose and insulin (to calculate HOMA), BTMs (BAP, P1NP, CTX-1, and TRAC5b) were measured. Bone characteristics were determined with pQCT at radius and tibia, and with DXA for central sites. Leptin, hs-CRP and HOMA correlated inversely with BTMs: the partial coefficients were 1.5–1.9 fold higher in males than in females. After adjusting for age, BMI, and other endocrine factors, leptin displayed an independent effect in males on radial bone mass (p = 0.019), tibial trabecular density (p = 0.025) and total hip BMD (p = 0.043), with lower densities in males with high leptin. In females, the model adjusting for age, BMI, and other endocrine factors, revealed that hs-CRP had independent effects on radial bone mass (p = 0.034) and lumbar spine BMD (p = 0.016), women with high hs-CRP having lower values. Partial correlations of adiponectin and TIMP-1 with bone characteristics were discrepant; MMP-8 showed no associations. In conclusion, in young obese adults and their controls, leptin, hs-CRP and HOMA associate inversely with BTMs and bone characteristics. Leptin appears to be the key independent effector in males, whereas hs-CRP displayed a predominant role in females.

## Introduction

Chronic inflammatory diseases and chronic inflammation are associated with bone loss and fragility fractures [1,2]. In general, factors that contribute to bone loss exert their effects by introducing a negative balance between bone formation and bone resorption. Preclinical studies provide compelling evidence on this matter. Moreover, chronic inflammation, induced by TNFα, inhibits osteoblastogenesis in various models [3]. Obese subjects have chronic low-grade systemic inflammation, which contribution to bone health has remained unclear.

High-sensitivity C-reactive protein (hs-CRP) is widely used as a marker of systemic low-grade inflammation. The association between hs-CRP and bone mineral density (BMD) or fracture risk has been at the scope of several studies [4–6]. Recent findings from the Tromsø Study indicate that elevated hs-CPR concentrations associate with higher BMI and age, lower physical activity (PA), and male gender [2]. Although an inverse association between hs-CRP and BMD was noted exclusively in men after adjusting for BMI, higher hs-CRP associated with increased fracture risk in both sexes suggesting that other, BMD-independent mechanisms may be involved. Chronic exposure to low-grade systemic inflammation from early age, as noted in childhood obesity, predisposes to cardiovascular morbidity [7,8]. Similar association may be true for skeletal complications. Abnormal metabolic milieu may affect bone mineral accrual and bone size [9,10]. In fact, Lucas et al. [11] demonstrated that high hs-CRP concentrations in overweight girls led to decreased BMD by 17 years of age.

Leptin, a pro-inflammatory cytokine produced by adipocytes, exerts central and peripheral actions on bone; in rodent models the overall effect appears beneficial for bone formation [12]. In contrast, we and others have proposed leptin to inhibit bone turnover in humans [13,14]. In fact, all markers of bone turnover are substantially lower in obese subjects compared with normal-weight controls. Insulin resistance may also play a role in these interactions, since there is a close connection between adipose tissue dysfunction and insulin resistance [7,15]. Insulin resistance is suggested to impair IGF-1 signaling which is vital for the muscle-bone unit [16]. This further emphasizes the negative impact of early obesity-related insulin resistance may have on bone health [16]. In line with this, several studies have suggested that insulin resistance in children results in impaired bone mass accrual [17,18].

High leptin concentrations, chronic low-grade inflammatory status, and insulin resistance often coexist in metabolically unhealthy obese subjects, who are at higher risk of developing type 2 diabetes. The unfavorable metabolic milieu may be the main culprit for increased fracture risk and impaired bone quality witnessed in obese subjects and patients with type 2 diabetes [19]. The aim of this study was to identify the drivers of obesity-related bone phenotype. Therefore, we have examined the associations of leptin, hs-CRP and insulin resistance with bone turnover markers (BTM) and bone characteristics measured with peripheral computed tomography (pQCT) and DXA in a cohort of young adults with morbid childhood-onset obesity and their population-based non-obese controls.

## Subjects and methods

### Subjects

This study was designed to assess skeletal and metabolic characteristics of severe childhood-onset obesity and was carried out at Children's Hospital, Helsinki University Hospital, Finland. An ethical approval was obtained from the Research Ethics Committee of the Hospital District of Helsinki and Uusimaa. Written informed consent was obtained from all study participants and in case of minors, the consent was obtained from their legal guardians as well. Inclusion criteria for the obese subjects were: i) weight-for-height ratio exceeding 60% before age 7 years, according to Finnish growth standards (comparable to BMI > 97^th^ percentile for age), ii) referral because of severe obesity to Children's Hospital, Helsinki University Hospital, during childhood, iii) residence in the capital region of Helsinki at age 7 years, and iv) aged between 15 and 25 years at the time of the study, as described earlier [10]. We identified a total of 366 patients fulfilling the inclusion criteria in the hospital's patient register and 68 (19%) of them consented to participate in the study. Control subjects were selected from the national population register based on their age and hospital catchment area (capital region of Helsinki). Controls were excluded if they had developed obesity (weight-for-height ratio above 40%) before age 10 years. A total of 73 controls consented to our study during 2011–2013.

### Methods

Anthropometry including height (cm), weight (kg), and waist (WC; cm) and hip circumferences (cm) was collected during the study visit and BMI (kg/m^2^) calculated, as described previously [10]. The health and diseases of the subjects, and their use of medicines were self-reported and collected with a questionnaire [10].

Areal bone mineral density (BMD_a_) for whole body (WB), lumbar spine (LS) and total hip (THIP), and WB fat percent (fat%) were measured with Lunar Prodigy Advance DXA (GE Healthcare, Madison, WI) in subjects with weight < 160 kg, thus data was not available for 8 obese subjects. Calibration of the measurement was performed with a spine phantom. Reducibility of DXA measurement for total body is: BMD = 0.85%, BMC = 0.45% and BA = 0.78% [20]. BMI, WC and fat% are referred to as obesity estimates in the text.

Bone characteristics of radius and tibia were examined with peripheral quantitative computed tomography (pQCT) (XCT-2000; Stratec; Pforzheim; Germany; software version 6.20). The radius was measured at distal (4%) and proximal (66%) sites and the tibia at distal (4%) and diaphyseal (33%) sites, as previously described [10]. For the present study the following variables were used: total bone mass (Mass; g/cm) and trabecular density (Trb Den; mg/cm^3^) from distal sites, cortical density (Crt Den; mg/cm^3^), polar strength strain index (SSIPOL; mm^3^) and periosteal circumference (PC; mm) from the proximal and diaphyseal sites. Scans were taken by two trained operators. The repeatability of the pQCT was evaluated with measurements of phantom provided by the manufacturer. For total, Trab and Cort cross-sectional area and density CV% were 0.24, 0.27; 0.25, 0.34; and 0.25, 0.31, respectively. Our in-house short-term precision (CV%) was determined with duplicate measurements of five subjects. CVs for the cross-sectional area and density in the total, Cort, and Trab bone were 1.91, 1.49; 3.0, 0.80; and 1.04, 1.0, respectively as described before [21].

#### Laboratory methods

Overnight fasting blood samples were obtained between 8.00 and 10.00 am for biochemistry. The samples were centrifuged after 30 minutes and serum was divided into aliquots and stored at– 80°C for further analyses.

Glucose was analysed by spectrophotometric hexokinase and glucose-6-phosphate dehydrogenase assay (Gluko-quant glucose/hexokinase, Roche Diagnostics) with a Hitachi Modular automatic. Serum insulin was measured with time-resolved immunofluorometric assay (Perkin Elmer Life Sciences, Finland) with a detection limit of 0.5 mU/l and an interassay-CV less than 4%. The insulin-resistance index determined by homeostasis model assessment (HOMA-IR) was calculated as the product of the fasting serum insulin concentration (in mU/l) and fasting plasma glucose concentration (in mmol/l) divided by 22.5. The glycosylated hemoglobin (HbA1c) was measured by photometric immunoassay.

Bone-specific alkaline phosphatase (BAP), intact N-terminal propeptide of type I collagen (PINP) and C-terminal cross-linked telopeptide of type I collagen (CTX-I), serum 25-OH vitamin D (S-25-OHD) and intact parathyroid hormone (iPTH) were measured with the IDS-iSYS automated analyzer (IDS Ltd, Boldon, UK), and tartrate-resistant acid phosphatase isoform 5b (TRACP 5b) using a manual assay (BoneTRAP^®^, IDS Ltd). 25-OHD concentrations by IDS-iSYS showed good linear agreement with liquid chromatography in tandem with mass spectrometry (LC-MS) (R^2^ = 0.942, in-house comparison performed with 67 samples). IDS-iSYS 25-OHD concentrations were 0.72-fold lower than those measured with LC-MS. Intra- and inter-assay CV% for 25-OHD were < 5% and < 8%, respectively. Our laboratory participates in the inter-laboratory quality assessment scheme for vitamin D, DEQAS.

Serum adiponectin was determined with Human Total Adiponectin/Acrp30 Quantikine ELISA Kit and serum leptin with Human Leptin R Quantikine ELISA Kit (R&D Systems, Minneapolis, USA) with intra- and inter-assay CV of <12%.

Matrix metalloproteinase 8 (MMP-8) levels from serum samples were measured by time-resolved immunofluorometric assay as described earlier [22, 23]. The interassay CV was 7.3% with a detection limit of 0.08 μg/l. Serum levels of tissue inhibitors of metalloproteinase 1 (TIMP-1) were measured with commercial enzyme-linked immunosorbent assay TIMP-1 Amersham ELISA (Human, Biotrak, ELISA system, GE Healthcare, Amersham, Buckinghamshire, UK) [23]. The interassay CV was 13.1% and the detection limit for this assay is 1.25 μg/l. The calculation of MMP-8/TIMP-1 molar ratio was performed as mol/l [23, 24]. hs-CRP was determined with immunoturbidimetric assay on Roche automated clinical chemistry analyzers at the central laboratory.

#### Statistical methods

Normality of the variables was visually inspected and logarithmic (BTMs, leptin, adiponectin, hs-CRP, HOMA, 25-OHD, PTH, MMP-8, TIMP-8, MMP-8/TIMP-1 molar ratio) transformations were made to obtain normal distribution. Pearson correlations were studied and confounding factors were identified for BTMs: age, gender and height and for bone characteristics: age, gender and BMI.

Based on our earlier findings [10] on gender difference, partial correlations between endocrine factors/obesity estimates and BTMs / bone characteristics were examined separately in males and females after adjustment for confounders. To dissociate independent effect of endocrine factors we compared BTMs and bone characteristics between groups of LOW and HIGH concentrations of the endocrine factors. Cut-off values for groups were defined as the median concentration of leptin, hs-CRP and HOMA in males and females separately. These analyses were performed with MANCOVA, where group mean values were adjusted for confounders, which included age, BMI, and other endocrine factors.

P values less than 0.05 were considered statistically significant. All statistical analyses were conducted using the IBM SPSS program for Windows version 22 (IBM, Chicago, IL, USA).

## Results

### Baseline characteristics

Complete data on circulating endocrine factors were available for 55 obese and 65 control subjects. The mean age of the participants was 19.5 (SD 2.5) years and 47.5% of them were males (Table 1). Obese subjects had greater weight, BMI and waist circumference, but similar height compared with controls. There were no reasons to exclude subjects based on their self-reported medical conditions. Obese subjects had higher concentrations of hs-CRP (4.5-fold), glucose, insulin (2.7-fold), leptin (4.7-fold) and PTH (1.5-fold), whereas concentrations of adiponectin (0.6-fold), 25-OHD (0.8-fold) and BTMs (except BAP) were lower than in control subjects. Concentrations of MMP-8 and TIMP-1, or their molar ratio did not differ between the groups. Impaired fasting glucose levels (= 6.1–6.9 mmol/l) were observed in 5 subjects (4.2%), while high fasting insulin concentrations (≥ 12 mU/l) were seen in 38 subjects (32%).

**Table 1:** Sex-specific baseline characteristics in obese subjects and their controls with mean (SD).

	Male		Female		P-value	
	Obese	Normal-weight	Obese	Normal-weight	for males	for females
N	28	29	27	36		
Age, y	19.1 (2.5)	19.8 (2.8)	19.1 (2.4)	19.9 (2.4)	0.296	0.173
Height, cm	179.7 (7.0)	180.5 (8.1)	167.7 (6.5)	166.9 (5.9)	0.674	0.604
Weight, kg	117.7 (29.7)	76.8 (13.9)	124.9 (27.9)	60.8 (7.3)	< 0.001	< 0.001
BMI, kg/m^2^	36.4 (9.2)	23.6 (4.3)	44.1 (8.2)	21.8 (2.3)	< 0.001	< 0.001
Waist circumference, cm	114.0 (19.5)	80.3 (9.7)	117.2 (18.7)	71.1 (7.1)	< 0.001	< 0.001
Total fat, %	40.6 (8.4)	20.0 (8.8)	52.1 (5.4)	32.0 (5.2)	< 0.001	< 0.001
Total fat, kg	46.1 (19.1)	15.5 (9.4)	59.5 (15.9)	18.7 (4.9)	< 0.001	< 0.001
hs-CRP, mg/l	3.3 (3.9)	0.9 (2.2)	8.2 (7.4)	1.7 (3.9)	0.007	< 0.001
B-HbA1c, mmol/mmol	36.8 (14.6)	34.1 (2.4)	36.7 (6.3)	33.4 (2.5)	0.328	0.007
fP-Glucose, mmol/l	5.6 (1.6)	5.3 (0.4)	5.4 (0.6)	5.0 (0.4)	0.327	0.002
fS-Insulin, mU/l	16.6 (11.8)	6.6 (3.5)	18.7 (11.2)	6.4 (3.6)	< 0.001	< 0.001
HOMA index	4.1 (3.1)	1.6 (0.9)	4.5 (2.7)	1.4 (0.8)	< 0.001	< 0.001
Leptin, pg/ml	27086 (20341)	4148 (4608)	60427 (25395)	13230 (6480)	< 0.001	< 0.001
Adiponectin, ng/ml	6114 (3475)	7188 (3754)	6820 (3001)	13394 (6476)	0.268	< 0.001
fP-PTH, ng/l	40.4 (22.2)	32.7 (19.6)	55.1 (28.7)	29.6 (16.6)	0.174	< 0.001
S-25-OHD, nmol/l	59.0 (25.6)	58.6 (17.5)	54.1 (18.2)	76.6 (27.9)	0.944	< 0.001
PINP, ng/ml	106.0 (50.0)	156.9 (108.0)	62.7 (25.5)	77.1 (33.4)	0.027	0.068
BAP, μg/l	27.5 (14.1)	31.8 (15.5)	19.7 (6.7)	16.8 (6.3)	0.286	0.083
CTX, ng/ml	0.97 (0.38)	1.28 (0.58)	0.61 (0.26)	0.73 (0.23)	0.021	0.053
TRACP5b, U/l	4.1 (1.2)	5.5 (1.7)	3.6 (1.0)	4.1 (1.0)	0.001	0.039
TIMP-1, ng/ml	39.7 (26.7)	44.5 (49.2)	40.4 (24.9)	28.9 (27.4)	0.665	0.090
MMP-8, ng/ml	138.3 (30.1)	139.9 (86.8)	117.2 (29.8)	118.6 (49.6)	0.930	0.897

Gender-specific differences were observed in HbA1c and fasting glucose, which were higher in obese women compared with normal-weight females (p = 0.007 and p = 0.002, respectively), while no differences were observed in males. In addition, obese females had higher PTH and lower 25-OHD and adiponectin than normal-weight females (p<0.001, for all), whereas these differences were not observed between obese and normal-weight males. On the other hand, of BTMs P1NP (p = 0.027) and CTX (p = 0.021) were lower in obese males compared with normal-weight males, whereas the differences between females did not reach formal statistical significance.

### Partial correlations with crude outcomes

Partial correlations for endocrine factors / obesity estimates and BTMs were investigated separately in males and females (Table 2). In general, the partial coefficients were 1.5- to 1.9-fold higher in males than in females, despite a similar number of subjects in the groups. This applied to leptin, hs-CRP, and HOMA, which were at the focus of our study. Leptin correlated inversely with BTMs except for BAP in both genders. hs-CRP showed consistent, inverse associations with all BTMs in males. These were similar, but weaker in females, except not for BAP. Interestingly, HOMA correlated inversely with BTMs except for BAP only in males. Of obesity estimates, BMI showed the strongest correlations with BTMs in both genders.

**Table 2:** Partial correlations between endocrine factors / obesity estimates and BTMs after controlling for age and height separately in males (n = 57) and females (n = 63).

		Ln(P1NP)	Ln(CTX)	Ln(BAP)	Ln/(TRACP5b)
Ln(Leptin)	m	-0.497*	-0.503**	-0.204	-0.532**
	f	-0.263*	-0.336*	0.195	-0.303*
Ln(hs-CRP)	m	-0.535**	-0.569**	-0.416*	-0.512**
	f	-0.284*	-0.377*	0.115	-0.298*
Ln(HOMA)	m	-0.371*	-0.468**	-0.135	-0.452**
	f	-0.107	-0.215	0.214	-0.084
Ln(Adiponectin)	m	0.326*	0.332*	0.204	0.315*
	f	0.184	0.170	-0.228	0.125
Ln(25-OHD)	m	0.155	0.075	-0.004	0.064
	f	0.022	0.091	-0.211	0.238
Ln(PTH)	m	-0.119	-0.108	-0.035	-0.462*
	f	-0.172	-0.204	0.041	-0.448**
Ln(MMP-8)	m	-0.263	-0.236	-0.115	-0.092
	f	0.050	0.077	0.272*	0.170
Ln(TIMP-1)	m	-0.305*	-0.127	-0.136	-0.305*
	f	0.045	0.010	0.160	0.070
BMI, kg/m^2	m	-0.605**	-0.684**	-0.358*	-0.595**
	f	-0.376*	-0.416**	0.175	-0.451**
WC, cm	m	-0.613**	-0.672**	-0.323*	-0.590**
	f	-0.355*	-0.408**	0.266*	-0.390**
Fat, %^1^	m	-0.524**	-0.543**	-0.208	-0.466**
	f	-0.351*	-0.384*	0.118	-0.382*

m; males, f; females,

*; p<0.05,

**; p<0.001

^1^n = 51 in males and n = 54 in females for Fat%

Strong cross correlations were observed between leptin and hs-CRP (r = 0.704, p < 0.001), leptin and HOMA (r = 0.575, p < 0.001) and hs-CRP and HOMA (r = 0.535, p < 0.001) in the age, sex and height adjusted model. Cross correlations of MMP-8 and MMP-8/TIMP-1 with leptin, hs-CRP and HOMA varied between 0.21 and 0.31 with p < 0.02 (S1 Table). Based on these results, an adjustment for BMI was justified in further analyses.

Partial correlations between metabolic factors / obesity estimates and bone characteristics are presented in Table 3 separately for males and females. The analyses were controlled for age and BMI. Leptin correlated inversely with Mass in both distal radius and tibia, and with Trb Den, and PC in tibia. Similarly, an inverse association was observed between leptin and BMD_a_ both in WB and THIP in males, but these were not present in females.

**Table 3:** Partial correlation between estimates of obesity/ endocrine factors and bone outcomes in age and BMI adjusted model separately for males and females.

		RADIUS					TIBIA					DXA		
		Mass, g/cm	Trb Den, mg/cm^3^	SSIPOL, mm^3^	Crt Den, mg/cm^3^	PC, mm	Mass, g/cm	Trb Den, mg/cm^3^	SSIPOL, mm^3^	Crt Den, mg/cm^3^	PC, mm	WB BMD, g/cm^2^	L1-L4 BMD, g/cm^2^	THIP BMD, g/cm^2^
Ln(Leptin)	m	-0.392*	-0.234	-0.200	-0.099	-0.222	-0.432*	-0.347*	-0.254	0.094	-0.363*	-0.352*	-0.254	-0.342*
	f	-0.027	0.070	-0.056	-0.084	-0.054	-0.100	0.037	-0.261	-0.140	-0.199	-0.116	0.039	-0.028
Ln(hs-CRP)	m	-0.122	0.005	-0.306*	-0.010	-0.224	-0.171	-0.090	-0.394*	0.319*	-0.488**	-0.210	-0.200	-0.180
	f	-0.401*	-0.320*	-0.136	0.260*	-0.335*	-0.415*	-0.293*	-0.359*	0.077	-0.390*	-0.374*	-0.240	-0.328*
Ln(HOMA)	m	-0.348*	-0.333*	-0.366*	-0.117	-0.304*	-0.382*	-0.295*	-0.266	-0.113	-0.293*	-0.394*	-0.372*	-0.362*
	f	-0.262*	-0.202	-0.068	0.016	-0.055	0.177	0.173	-0.006	-0.201	0.078	0.017	0.311*	0.105
Ln(Adiponectin)	m	-0.076	-0.247	0.218	0.069	-0.038	-0.044	-0.170	0.206	0.058	0.225	-0.008	0.099	-0.084
	f	0.015	-0.161	0.126	-0.108	0.059	0.189	0.078	0.321*	-0.324*	0.404*	-0.022	-0.088	-0.100
Ln(25-OHD)	m	0.370*	0.089	0.169	0.083	0.211	0.357*	0.227	0.167	0.117	0.248	0.332*	0.273*	0.309*
	f	-0.042	0.007	-0.129	-0.073	-0.103	0.112	0.239	-0.111	-0.215	-0.007	-0.020	-0.018	-0.008
Ln(PTH)	m	-0.093	-0.073	-0.033	-0.029	-0.201	-0.102	-0.047	0.026	0.019	-0.132	-0.082	0.022	-0.123
	f	-0.209	0.060	-0.213	-0.132	-0.126	-0.196	0.098	-0.288*	0.024	-0.271	-0.046	-0.241	0.049
Ln(MMP-8)	m	0.147	0.060	0.064	-0.060	0.092	0.152	-0.016	-0.098	0.207	-0.053	-0.040	0.072	0.037
	f	0.171	-0.018	0.094	0.009	0.113	0.032	-0.062	0.114	0.109	0.103	0.048	0.082	-0.041
Ln(TIMP-1)	m	0.193	0.100	-0.061	0.123	-0.045	0.039	0.054	0.059	0.196	-0.065	0.244	0.156	0.278
	f	0.165	0.108	0.156	-0.012	0.077	0.011	-0.085	-0.075	0.294*	-0.134	0.106	0.038	0.007
WC, cm	m	-0.133	-0.169	-0.068	-0.024	0.078	-0.120	-0.287*	0.241	0.110	0.147	-0.044	0.024	-0.122
	f	-0.030	-0.234	-0.050	0.040	0.093	-0.042	-0.137	0.105	-0.122	0.081	-0.290*	-0.154	-0.230
Fat, % ^1^	m	-0.365*	-0.211	-0.281*	-0.037	-0.238	-0.374*	-0.257	-0.070	0.209	-0.158	-0.213	-0.077	-0.209
	f	-0.359*	-0.502**	0.011	0.058	-0.196	-0.073	-0.071	-0.180	0.312*	-0.230	-0.223	-0.026	-0.166

m; males, f; females,

*; p<0.05,

**; p<0.001

^1^n = 51 in males and n = 54 in females for Fat%

The data on the use of medicines were not available in 2 female subjects. In total 6 male and 4 female subjects reported the use of inhaled glucocorticoids: 5 for asthma, and others for non-specific difficulties in breathing or allergy-related symptoms. This did not differ between obese or normal-weight subjects, neither in males (2/28 vs. 4/29, p = 0.670) nor in females (2/26 vs. 2/35, p = 0.999), and was not related to BTMs (data not shown).

hs-CRP correlated inversely with SSIPOL both in radius and tibia, and with PC of tibia in males. In females, inverse associations of hs-CRP were observed with Mass, Trb Den and PC in radius and tibia and along with SSIPOL in tibia. In addition, hs-CRP correlated inversely with BMD_a_ in WB and THIP in females. On the contrary, positive associations were noted between hs-CRP and radial Crt Den in females and between hs-CRP and tibial Crt Den in males.

HOMA correlated inversely with various bone characteristics (Mass, Trb Den, SSIPOL, PC, BMD_a_ in WB, LS and THIP) in males. Of these, only an inverse association with radial Mass and positive association with LS BMD_a_ were present in females.

In males, adiponectin was not associated with bone outcomes, but in females positive associations were demonstrated with SSIPOL and PC in tibia, while inverse with Crt Den. TIMP-1 showed a positive correlation with Crt Den only in females, but not in males. No association were present between MMP-8/ MMP-8/TIMP-1 and bone characteristics.

### Comparison between LOW and HIGH groups

To understand the drivers of obesity-related bone phenotype we investigated independent effects by comparing BTMs and bone characteristics between LOW and HIGH groups defined by median concentration of the metabolic factor in males and females (Table 4).

**Table 4:** Difference in bone outcomes with SEM between HIGH and LOW median groups after adjustment in males and females.

		Radius		Tibia		BTM		DXA	
	Sex	ΔHIGH-LOW	SEM	ΔHIGH-LOW	SEM	ΔHIGH-LOW	SEM	ΔHIGH-LOW	SEM
		Mass, g/cm		Mass, g/cm		Ln(P1NP)		WB BMD, g/cm^2^	
Leptin^1^	m	-0.157	0.103	-0.562*	*0*.*255*	0.108	0.162	-0.073	0.042
	f	-0.013	0.052	-0.054	0.137	-0.053	0.138	-0.030	0.021
hs-CRP^2^	m	0.064	0.088	-0.111	0.221	-0.099	0.146	0.000	0.038
	f	-0.096*	*0*.*044*	-0.138	0.103	-0.012	0.105	-0.026	0.019
HOMA^3^	m	0.023	0.095	0.023	0.246	-0.192	0.143	-0.015	0.041
	f	-0.090	0.051	0.047	0.127	0.017	0.902	-0.009	0.023
		Trb Den, mg/cm^3^		Trb Den, mg/cm^3^		Ln(CTX)		LS BMD, g/cm^2^	
Leptin^1^	m	-20.730	15.226	-34.644*	*15*.*319*	0.060	0.137	-0.089	0.063
	f	-8.739	11.067	-8.836	11.827	-0.114	0.134	-0.025	0.040
hs-CRP^2^	m	5.306	13.503	-11.694	13.286	-0.169	0.118	-0.003	0.057
	f	-14.283	9.339	-11.329	8.733	-0.020	0.105	*-0*.085*	*0*.*034*
HOMA^3^	m	-6.839	14.710	3.550	14.702	*-0*.272*	*0*.*114*	-0.081	0.061
	f	0.109	11.155	14.190	11.026	-0.122	0.340	0.040	0.045
		SSIPOL, mm^3^		SSIPOL, mm^3^		Ln(BAP)		THIP BMD, g/cm^2^	
Leptin^1^	m	14.247	31.454	-96.717	154.776	0.286	0.177	-0.144*	*0*.*066*
	f	1.856	23.752	-116.353	102.025	0.211	0.128	-0.027	0.035
hs-CRP^2^	m	0.738	28.469	-51.554	137.613	-0.054	0.163	0.052	0.059
	f	-0.392	19.608	-56.028	74.504	0.090	0.100	-0.046	0.031
HOMA^3^	m	-33.707	29.728	46.819	140.765	-0.160	0.158	-0.015	0.064
	f	8.454	23.385	-89.207	93.679	0.093	0.443	-0.036	0.038
		Crt Den, mg/cm^3^		Crt Den, mg/cm^3^		Ln(TRACP5b)			
Leptin^1^	m	-13.563	11.446	-2.787	8.674	0.197	0.114		
	f	-4.524	10.201	10.210	8.170	0.127	0.085		
hs-CRP^2^	m	-5.359	10.062	5.552	7.621	-0.030	0.104		
	f	6.062	8.620	-1.348	6.052	0.015	0.066		
HOMA^3^	m	9.536	10.438	1.956	7.685	-0.040	0.105		
	f	-11.882	9.648	1.110	7.914	0.108	0.187		
		PC, mm		PC, mm					
Leptin^1^	m	1.080	1.443	-1.281	1.931				
	f	-0.555	1.208	-0.840	1.729				
hs-CRP^2^	m	0.022	1.301	-2.279	1.723				
	f	-1.210	1.035	-0.403	1.270				
HOMA^3^	m	-1.486	1.378	0.888	1.747				
	f	0.622	1.173	-0.496	1.612				

BTM; bone turnover markers, ΔHIGH-LOW; difference between HIGH and LOW, m; male, f; female,

*p < 0.05

^1^in MANOVA covariates: age, BMI, Ln(hs-CRP), Ln(HOMA) and Ln(Adiponectin)

^2^in MANOVA covariates: age, BMI, Ln(Leptin), Ln(HOMA) and Ln(Adiponectin)

^3^in MANOVA covariates: age, BMI, Ln(Leptin), Ln(hs-CRP) and Ln(Adiponectin)

Although leptin demonstrated no independent effect on BTMs nor radial characteristics, males with HIGH LEPTIN had lower Mass (adjusted mean values for LOW and HIGH groups were 4.267 vs. 3.708 g/cm, p = 0.019, respectively) and lower Trb Den (260 vs. 226 mg/cm^3^, p = 0.025) in tibia than males with LOW LEPTIN. In parallel with this finding, THIP BMD_a_ differed by leptin group: males with HIGH leptin having lower BMD_a_ than males with LOW LEPTIN (1.209 vs. 1.065 g/cm^2^, p = 0.043).

After adjusting for other endocrine factors, hs-CRP showed no independent effect on BTMs nor tibial outcomes in either gender. However, in females radial Mass differed by hs-CRP: women with HIGH hs-CRP had a lower Mass compared with women with LOW hs-CRP (1.159 vs 1.063.g/cm, p = 0.034), but this was not seen in males. Moreover, females with HIGH hs-CRP showed also lower LS BMD_a_ compared with LOW hs-CRP group (1.204 vs 1.155 g/cm^2^, p = 0.016)

An independent effect was observed also for HOMA, but only in males. Males in HIGH HOMA group had lower Ln(CTX) (0.147 vs. -0.110 p = 0.029) than males in LOW HOMA group, but this did not apply to other BTMs or any of the bone characteristics.

To summarize, key drivers of obesity-related bone phenotype were sex-specific: significant independent effects of leptin on bone characteristics were marked in males, while in females hs-CRP displayed an independent role.

As delayed pubertal development may have impacted the results especially in males, we performed a sensitivity analysis focusing on boys aged at least 17 years (n = 43), who were considered to be at a late stage of pubertal development. In the sensitivity analysis, the results concerning leptin were repeated: males with HIGH LEPTIN had lower Mass (4.370 vs. 3.694 g/cm, p = 0.042, for LOW and HIGH groups, respectively) and lower Trb Den (265 vs. 226 mg/cm^3^, p = 0.050) in tibia than males with LOW LEPTIN. Correspondingly, THIP BMD_a_ differed by leptin group: males with HIGH leptin having lower BMD_a_ than males with LOW LEPTIN (1.235 vs. 1.045 g/cm^2^, p = 0.023). The results concerning HOMA and BTMs were similar, but lacked the formal statistical significance: males in HIGH HOMA group had lower Ln(CTX) (0.018 vs. -0.152 p = 0.074) than males in LOW HOMA group.

## Discussion

The main finding in the present study is that in obese young adults HOMA, leptin and hs-CRP associate inversely with bone turnover markers and bone characteristics both in peripheral and central skeleton. These associations are more apparent in males, in line with our previous finding that early-onset obesity is more harmful for the bone strength in males [10].

We observed consistent inverse associations between multiple endocrine factors and P1NP/ CTX/ TRACP5b. However, there were no discordant effects on markers of bone formation and bone resorption. There might be several explanations for this, but the most evident one seems to be coupled bone turnover: when inflammatory status decreases bone formation, the secondary observed effect will be impaired bone resorption, or vice versa. In fact, formation and resorption markers demonstrated strong positive correlations with each other (r between 0.6 and 0.8, p<0.001, in the whole group, data not shown). The proposed mechanism might involve suppression of Wnt signaling by sclerostin [25].

Leptin is an adipocyte-derived cytokine that closely reflects the amount of body fat, while hepatocyte-originated hs-CRP reflects systemic low-grade inflammation typically initiated by pro-inflammatory factors. In our cohort leptin and hs-CRP showed the strongest correlations both with BTMs and peripheral and central bone characteristics in both males and females. However, independent effects of leptin were observed only in males, while in females hs-CRP showed independent effects. The pro-inflammatory cytokine leptin is proposed to suppress both bone formation and resorption [14,26]. The long-term skeletal consequences may be similar to what was observed here, with males having lower total bone mass and trabecular density in tibia and areal BMD in the hip.

Obesity is characterized by low-grade inflammation, as reflected by elevated levels of leptin and hs-CRP. Unsurprisingly, we observed relatively strong cross-correlations between leptin and hs-CRP. Adipocytes produce and secrete IL-6, which upregulates CRP synthesis in hepatocytes [27]. More recent in vivo data suggest that also leptin promotes CRP synthesis in the liver as well as in vascular/endothelial cells [28]. In turn, CRP may be able to regulate circulating leptin bioavailability by binding to it, which impairs the ability of both leptin and CRP to bind to their respective receptors [28]. Taken together, there appears a close and complex interplay between leptin and CRP. In males, independent effects of leptin were marked in several bone characteristics, while in females hs-CRP appeared predominantly. In our cohort, females were more severely obese than males, with 2.5-fold higher hs-CRP concentrations, which might explain some of the observed differences.

Insulin resistance affects bone cells and impairs bone formation most likely by inhibiting Wnt/beta-catenin signaling [29]. In rodent models, obesity accompanied with insulin resistance is demonstrated to decrease osteoblastic proliferation, increase osteoblastic apoptosis, enhance osteoblastic insulin resistance and increase bone porosity [30] which are marked as impaired bone quality especially in trabecular bone sites. In the present study, inverse associations of HOMA with P1NP, CTX, TRAC5b and multiple bone characteristics were observed almost exclusively in males. However, an independent effect of HOMA was verified only in CTX: with increased insulin resistance, the bone resorption was impaired in males. This finding is hard to interpret since the prevalence of insulin resistance did not differ between obese males and females. Our finding is in line with the European Male Ageing Study reporting HOMA to correlate independently and inversely with BTMs; P1NP and CTX [31]. During normal bone remodeling resorption and formation are coupled. In our study the findings on P1NP are supportive, but non-significant. A close relationship between obesity-induced low-grade inflammation and insulin resistance is reported [32]. In the present study 32% of all subjects and 64% of obese subjects were insulin resistant. Given that our study population was young, it may have been premature to study the associations between insulin resistance and bone characteristics. It is possible that longer exposure to insulin resistance may be needed to observe consistent associations. Clinical findings in pediatric populations suggest insulin resistance to precede bone maturation [18] and impair muscle-bone unit development [16]. This is supported by recent animal data showing that hyperglycemia reduces responses to mechanical loading [33].

Despite the predominantly inverse associations, also positive associations were noted between fat% / hs-CRP / TIMP-1 and cortical density. This illuminates the controversy of our topic [19]; obesity and early stages of diabetes including hyperinsulinemia may also have anabolic effects on bone. Of the bone characteristics, bone mass and trabecular density are the most susceptible to the alterations in the metabolic milieu [31]. Evidently, the more abundant vascularization of trabecular bone compartment compared with cortical bone and in general more rapid bone turnover may partly explain our findings, while for cortex properties the loading plays a crucial role. Our findings are in accordance with [34] Romagnoli et al. in reporting lower trabecular bone score in lumbar spine in obese men compared with overweight men.

Our study has some limitations. The number of subjects was relatively low and the study was of cross-sectional nature. However, we had comprehensive data including various BTMs, pQCT and DXA characteristics and numerous endocrine factors. Estrogen and testosterone concentrations were not available. Similarities have been suggested between estrogen and leptin in influencing bone remodeling [12]. The relationship between leptin and BMD may be age dependent [35], but leptin is reported to associate inversely with cortical thickness and cross-sectional area in tibia in young adult males [36]. Information on pubertal status was not collected. As delayed pubertal development may have impacted the results especially in males, we performed a sensitivity analysis focusing on boys aged at least 17 years. Since the sensitivity analysis replicated our findings, it is unlikely that different growth pattern between girls and boys would have explained the observed differences between genders. The primary focus of our study was solely on metabolic milieu and often it results from sedentary lifestyle and unhealthy diet in obese subjects. The strength of the present study is that we included both sexes and subjects with varying BMI and in statistical analyses we adjusted for these factors. Thus, our finding could be applied widely to all young subjects.

In conclusion, in a cohort of young obese adults and their controls leptin, hs-CRP and HOMA associated inversely with BTMs and bone characteristics. The key drivers were sex-specific: in men the independent effects of leptin were most prominent on total bone mass, trabecular BMD in radius and THIP BMD_a_. In females, independent effects of hs-CRP were discovered in radial bone mass and LS BMD_a_. Obesity is characterized with co-existing metabolic disturbances, and of these especially leptin in males and hs-CRP in females associate with impaired skeletal health.

## Supporting information

S1 Table Partial cross-correlations between endocrine factors / obesity estimates after controlling for age, gender and height. (DOCX)
