# Supplementary material for: Metabolic milieu associates with impaired skeletal characteristics in obesity
Source: PLoS One. 2017 Jun 22;12(6):e0179660. doi: 10.1371/journal.pone.0179660 (PMC5480955; doi:10.1371/journal.pone.0179660)
Supplement: S1 Table — (DOCX) [file pone.0179660.s001.docx]

S1 Table. Partial cross-correlations between endocrine factors / obesity estimates after controlling for age, gender and height.
